# Clinical presentations and treatment outcomes of Hashimoto encephalopathy at Siriraj Hospital – Thailand’s largest national tertiary referral center

**DOI:** 10.1186/s12883-023-03305-4

**Published:** 2023-09-22

**Authors:** Chaisak Dumrikarnlert, Smathorn Thakolwiboon, Vorapun Senanarong

**Affiliations:** 1 Division of Neurology, Department of Medicine, Faculty of Medicine Siriraj Hospital, Mahidol University, Bangkok, Thailand; 2 Neuroscience Center, Bangkok International Hospital, Bangkok, Thailand

**Keywords:** Clinical presentations, Treatment outcomes, Hashimoto encephalopathy, Siriraj Hospital, Thailand

## Abstract

**Background:**

Hashimoto encephalopathy has multiple clinical presentations, and other than the presence of thyroid antibody, laboratory and imaging investigations are all non-specific. Data specific to the clinical presentations and treatment outcomes of patients with Hashimoto encephalopathy in Thailand remain scarce.

**Objectives:**

To retrospectively investigate the clinical presentations and treatment outcomes of patients with Hashimoto encephalopathy at Siriraj Hospital.

**Methods:**

Patients who presented with acute encephalopathy at our center during July 2012-March 2017 were evaluated for eligibility. The inclusion criteria were positive anti-thyroperoxidase (anti-TPO) or anti-thyroglobulin (anti-Tg) in serum with negative neuronal antibody in serum or cerebral spinal fluid (CSF). Clinical presentations, symptom duration, laboratory results of thyroid status and thyroid autoantibody, CSF study, and clinical outcomes were collected.

**Results:**

Of the 204 patients who presented with encephalopathy, 31 (15.2%) were positive for the anti-TPO or anti-Tg antibody. Of those, 13 patients met the diagnostic criteria for Hashimoto encephalopathy. Clinical presentations included cognitive impairment (76.9%), clouding of consciousness (46.2%), and behavior change (30.8%). The neuropsychiatric presentations were visual hallucination (30.8%), auditory hallucination (15.4%), delusion (7.7%), and mood disturbance (23.1%). Other clinical presentations included seizure (38.5%), abnormal movement (23.1%), sleep disturbance (38.5%), ataxia (46.2%), stroke-like episode (15.4%), and fever (15.4%). Most patients (76.9%) had onset within < 3 months. Regarding outcomes, 1 patient who did not receive corticosteroid died from status epilepticus and septic shock. Among the 12 patients who received corticosteroid, 9 (75%) had marked improvement, 1 (8.3%) had slight improvement, and 2 (16.6%) had no clinical improvement. Seven patients (53.9%) had normal thyroid function, 4 patients (30.8%) had subclinical hypothyroidism, and 2 patients (15.4%) had subclinical hyperthyroidism.

**Conclusions:**

The results of this study revealed cognitive impairment, neuropsychiatric symptoms, seizure, ataxia, and sleep disturbance to be common manifestations of Hashimoto encephalopathy. This condition should always be considered in individuals with subacute onset of unexplained cognitive impairment or cerebellar ataxia. Laboratory and neuroimaging investigations were all found to be nonspecific in Hashimoto encephalopathy. Most patients responded well to treatment, so clinical suspicion and early diagnosis and treatment will lead to improved patient outcomes.

**Supplementary Information:**

The online version contains supplementary material available at 10.1186/s12883-023-03305-4.

## Introduction

Hashimoto encephalopathy, which was previously referred to as steroid responsive encephalopathy associated with autoimmune thyroiditis (SREAT), is an uncommon syndrome with many different clinical presentations [1]. Its clinical features can vary from mild (e.g., confusion) to severe (e.g., coma) [2–4]. The fact that it has a wide range of clinical presentations accompanied by normal or nonspecific magnetic resonance imaging (MRI) brain and cerebral spinal fluid (CSF) findings, diagnosis relies mainly upon clinical suspicion [5–8].

Hashimoto encephalopathy is thought to be an immune-mediated disorder, so the mainstay of treatment is immunosuppressive drugs, especially corticosteroid [1, 9]. Most patients respond well to steroid treatment; however, the patients who cannot tolerate the side effects of steroid treatment require other immunosuppressive agents [9–11]. Patients who present with seizure may also require short-term treatment with antiepileptic agents. Although the disease responds well to corticosteroid therapy in most patients, recovery is another matter. A previously published case series reported complete recovery or partial neurological response in 93% of patients [9]. However, 16% of patients who were followed over a median follow-up time of 12 months that disease relapse [9], and some patients required long-term immunosuppressant [11].

Given the scarcity of data specific to the clinical presentations and treatment outcomes of patients with Hashimoto encephalopathy in Thailand, the aim of this study was to retrospectively investigate the clinical presentations and treatment outcomes of patients with Hashimoto encephalopathy at Siriraj Hospital – Thailand’s largest national tertiary referral center.

## Methods

### Study design and population

Patients who presented with acute encephalopathy at our center during July 2012 to March 2017 were evaluated for eligibility. The inclusion criteria were, as follows: (1) Positive anti-thyroperoxidase (anti-TPO) or anti-thyroglobulin (anti-Tg) antibody; (2) Euthyroid or subclinical hypo/hyperthyroidism status; (3) Negative neuronal antibodies in serum or CSF; and, (4) Normal brain MRI or having non-specific abnormalities. The exclusion criteria were (1) History of drugs or substances that cause encephalopathy, and (2) having other known causes of encephalopathy from brain imaging, such as acute stroke.

Collected data included clinical presentations, duration of symptoms prior to diagnosis, laboratory results of thyroid status and thyroid autoantibody (anti-Tg, anti-TPO), CSF study, neuroimaging, and clinical outcome as measured by Modified Ranking Scale (mRS) at 3 months after treatment. We selected mRS for measurement of clinical outcome because it is the same tool that we use at our center to evaluate the treatment outcome of neurological patients, such as those who suffered a stroke. After treatment with steroid, we defined response as no improvement if there was no change in mRS, slight improvement if the mRS decreased by 1, and marked improvement if the mRS decreased by more than 1.

Glasgow coma scale [12] or Confusion Assessment Method [13] were used to evaluate consciousness of subject. Clouding of consciousness were defined by any abnormalities in either of both scores. CSF study consists of cell count, cell differentiate, protein, sugar, amyloid beta 42, total tau, phosphorylated tau, autoimmune encephalitis antibody panel, and paraneoplastic antibody panel. Antibodies in autoimmune encephalitis and paraneoplastic panel in our study are NMDA, AMPAR, CASPR2, LGI1, Hu, Ri, Yo, Ma, CRMP5, Amphiphysin, GAD.

## Results

**Fig. 1:**
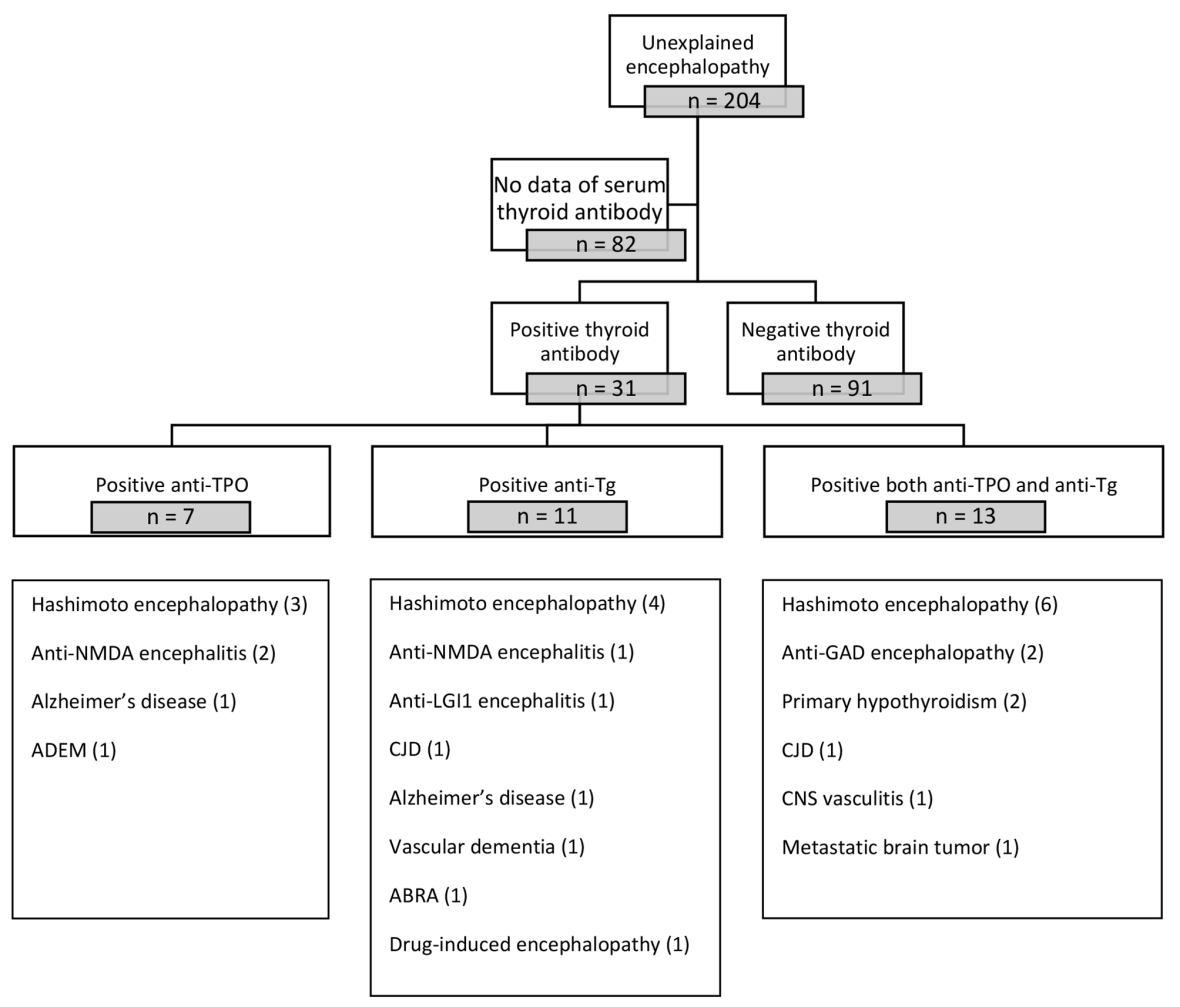
Flow diagram and study population by thyroid antibody Note: ADEM – Acute Disseminated Encephalomyelitis; CJD – Creutzfeldt-Jakob Disease; ABRA – Amyloid-Beta Related Angiitis; CNS – Central nervous system; NMDA – N-methyl-D-aspartate; GAD – Glutamic Acid Decarboxylase; LGI1 – Leucine-rich glioma-inactivated 1

In duration of follow up from 6 months up to 5 years there are 204 patients who presented at our center with unexplained acute encephalopathy, 31 (15%) tested positive for the anti-TPO or anti-Tg antibody. A total of 91 (44%) patients tested negative for both antibodies, and the remaining 82 (40%) patients had no antibody testing data in their medical chart. Among those with positive antibody test results, 13 patients met the diagnostic criteria for Hashimoto encephalopathy [14]. Among those 13 patients, the median age at onset was 63 years (range: 22–83) and 4 (30.7%) patients were female. Three (23.1%) patients tested positive for the anti-TPO antibody only, 4 (30.8%) tested positive for the anti-Tg antibody only, and 6 (46.1%) tested positive for both the anti-TPO and anti-Tg antibodies. Flow chart of our study population and diagnosis in each group categorized by thyroid antibody was shown in Fig. 1.

The clinical presentation of encephalopathy in these 13 patients was cognitive impairment in 10 (76.9%) patients, clouding of consciousness in 6 (46.2%) patients, and behavior change in 4 (30.8%) patients. Neuropsychiatric symptoms were found in 6 patients. Of those, 4 (30.8%) patients had visual hallucination, 2 (15.4%) patients had auditory hallucination, 1 (7.7%) patient had delusion, and 3 (23.1%) patients had mood disturbance. Seizure was found in 5 (38.5%) patients, and 3 (23.1%) of those had status epilepticus. Other clinical presentations were abnormal movement in 3 (23.1%) patients, sleep disturbance in 5 (38.5%) patients, ataxia in 6 (46.2%) patients, stroke-like episode in 2 (15.4%) patients (presented with recurrent episode of amaurosis fugax), and fever in 2 (15.4%) patients. Onset of symptoms was less than 3 months in 10 (76.9%) patients, 3–6 months in 2 (15.4%) patients, and 7 months in 1 (7.7%) patient. A summary of the clinical presentations among our 13-patient cohort is shown in Table 1.

Regarding thyroid status, we found 7 (53.8%) patients with euthyroidism, 4 (30.8%) patients with subclinical hypothyroidism, and 2 (15.4%) patients with subclinical hyperthyroidism. Physical examination of thyroid gland in 13 patients were all within normal limits, no tenderness or palpable mass and had normal size. Concerning other laboratory findings, 2 of 6 patients who were checked for antinuclear antibody (ANA) had positive titer, 3 patients had low vitamin D level, C-reactive protein (CRP) was abnormally high in 1 patient, and erythrocyte sedimentation rate (ESR) was abnormally high in 5 patients. CSF analysis revealed pleocytosis in 2 (15.3%) patients, and elevated protein in 6 (46.1%) patients. A summary of laboratory findings is shown in Table 2.

MRI brain with gadolinium injection, which was the neuroimaging study performed in all 13 patients in our study, revealed non-specific white matter change in an area or areas that is/are nonspecific to any symptoms, such as corona radiata. None of the lesions in any of our 13 patients were compatible with a diagnosis of multiple sclerosis (MS), neuromyelitis optica (NMO), previous stroke, or brain injury.

Of those 13 patients, 1 patient who did not receive corticosteroid died from status epilepticus and septic shock. Of the remaining 12 patients who did receive corticosteroid treatment, 9 patients (75%) had marked improvement, 1 patient (8.3%) had slight improvement, and 2 patients (16.6%) had no clinical improvement.

**Table 1:** Summary of the clinical presentations of 13 Thai patients diagnosed with Hashimoto encephalopathy

Clinical features	Number of patients	Percentage (%)
Encephalopathy - Clouding of consciousness - Cognitive impairment - Behavior change	13 6 10 4	100% 46.2% 76.9% 30.8%
Neuropsychiatric symptoms - Visual hallucination - Auditory hallucination - Delusion - Mood disturbance	6 4 2 1 3	46.2% 30.8% 15.4% 7.7% 23.1%
Seizure - Non-status epilepticus - Status epilepticus	5 2 3	38.5% 15.4% 23.1%
Abnormal movement (myoclonus)	3	23.1%
Sleep disturbance - Insomnia - Hypersomnolence	5 2 3	38.5% 15.4% 23.1%
Ataxia	6	46.2%
Stroke-like episode (blindness)	2	15.4%
Fever	2	15.4%
Duration of symptom onset		
- Less than 3 months - 3–6 months - More than 6 months	10 2 1	76.9% 15.4% 7.7%

**Table 2:** Summary of the laboratory findings among 13 Thai patients diagnosed with Hashimoto encephalopathy

Laboratory findings	Number of patients	Percentage (%)
Serum autoantibody		
- Anti-TPO positive - Anti-Tg positive - ANA positive	9/13 10/13 2/6	69.2% 76.9% 33.3%
Thyroid status		
- Euthyroid - Subclinical hypothyroid - Subclinical hyperthyroid	7/13 4/13 2/13	53.8% 30.8% 15.4%
CSF findings		
- CSF pleocytosis - CSF protein elevation	2/13 6/13	15.4% 46.2%

Abbreviations: TPO, thyroperoxidase; Tg, thyroglobulin; ANA, antinuclear antibody; CSF, cerebrospinal fluid

## Discussion

Hashimoto encephalopathy is a rare disease that may be associated with Hashimoto thyroiditis [1]. The clinical characteristics of this disease include subacute onset of encephalopathy, seizure, and abnormal movement (mostly myoclonus) [2, 3]. Approximately 25% of Hashimoto encephalitis patients present with stroke-like pattern of multiple, recurrent, acute to subacute episodes of focal neurological deficit[15]. Due to the variety and non-specificity of clinical presentations, diagnosis is challenging and is largely dependent upon clinical suspicion. A recently published study categorized the presenting syndromes of Hashimoto encephalopathy, as follows: psychiatric, encephalopathy, new-onset refractory status epilepticus, and limbic encephalitis [16, 17]. Similar to previous reports, the most common presenting symptom in the present study was cognitive impairment (76.9%) followed by stroke-like episode (15.4%) [2, 3, 15]. In contrast to previous studies that reported isolated psychiatric symptoms in 10–25% of patients [9, 16], we had no patients with isolated psychiatric symptoms. This finding may be explained by the fact that we included only patients who were negative for neuronal antibody. Moreover, previous study reported that approximately 80% of patients who presented with isolated psychiatric illness were not tested for neuronal surface antibody [9]; however, we now know that other autoimmune encephalitis can present with psychiatric manifestations [18–21]. Therefore, a diagnosis of Hashimoto encephalopathy that is based on isolated psychiatric symptoms alone should not be made with any degree of certainty or confidence, and more research is needed to better understand the relationship between psychiatric manifestations and Hashimoto encephalopathy.

Similar to previous reports, disease onset in most patients in our study was within 3 months [2, 22], but 3 patients had disease onset lasting longer than 3 months. These patients had comparatively minor and non-specific symptoms, which explains why they took a longer time to seek medical care. Those 3 patients first presented with slowly progressive cognitive decline with psychomotor retardation, but their symptoms did not markedly disturb their ability to perform normal activities of daily living. However, they later developed myoclonus and ataxia, which alerted them to the need to seek medical care. The 2 patients with stroke-like symptom also presented with recurrent episode of amaurosis fugax. After hospitalization, both of those two patients developed encephalopathy. Investigations revealed only an abnormality in thyroid antibody, so Hashimoto encephalopathy was suspected. Previous studies reported focal neurological deficits in Hashimoto encephalopathy to be weakness, numbness, diplopia, sensory disturbance, dysarthria, and gait disturbance [19–25]. The signs and symptoms prompting suspicion of Hashimoto encephalopathy in this setting were multiple recurrent episodes of focal neurological symptoms with varying degrees of cognitive dysfunction and alteration of consciousness. As such, a presentation of stroke-like symptom alone may be insufficient for making a definitive diagnosis of Hashimoto encephalopathy, so continued observation for other clinical manifestations is needed.

Previous studies [9, 13, 26] reported thyroid hormone status in Hashimoto encephalopathy to be euthyroidism in 18–45% of patients; however, we found a higher prevalence of 53%. In similar contrast, we found no cases of hypothyroidism in our study; however, the immediately aforementioned studies reported a prevalence of hypothyroidism of 17–30%. This difference in the proportion of cases with hypothyroidism may be due to our study’s small sample size. The same reason may also explain why we found a prevalence of hyperthyroidism if 15%, which is substantially higher than the 7% rate reported in the literature. ANA was screened in 6 of 13 patients, and 2 of those 6 patients (33.3%) had positive titer. Both patients demonstrated a nucleolar pattern, and the titer was borderline in one patient, and 1:100 in the other patient. Neither patient had clinical or other laboratory abnormalities that fulfill the diagnostic criteria for systemic lupus erythematosus (SLE), systemic sclerosis, or Sjӧgren syndrome. Even though autoimmune origin has been hypothesized in the pathogenesis of Hashimoto encephalopathy, and it was reported that 30% of patients also had an accompanying autoimmune disease [27], we found no evidence of this in our study. This disparity in findings may be due to our small sample size and/or the short duration of disease monitoring in our study.

Even though CSF analysis and neuroimaging results are both nonspecific to Hashimoto encephalopathy, they are both essential for ruling out other diseases, such as meningoencephalitis, paraneoplastic or autoimmune encephalitis, stroke, and vasculitis [13, 28]. Approximately 50% of MRI findings in Hashimoto encephalopathy reported as normal and remaining MRI brain imaging show non-specific lesion such as, generalized brain atrophy or increase signal in subcortical white matter area on T2-weighted/Fluid attenuated inversion recovery (FLAIR) sequence [29, 30]. Six patients had mild elevation of protein in CSF without detection of white blood cells (WBC) or low sugar, which is nonspecific. In the 2 patients in whom pleocytosis was detected, their WBC in CSF was less than 10 and the CSF of both patients had normal protein and sugar so infection was not considered to be a likely cause of their abnormalities. Since previous studies [2, 9, 26] reported that mild elevation of protein can be observed in the CSF of approximately 82%, and lymphocytic pleocytosis can be found 10–25% of Hashimoto encephalopathy patients, we postulated a diagnosis of Hashimoto encephalopathy in these patients.

The more specific biomarker than thyroid antibodies or neuroimaging that had been interested in recent years is autoantibody against the Amino (NH2)-Terminal of α-Enolase (NAE). High titers of this antibodies had been found in serum and CSF of Hashimoto encephalopathy and considered a potential biomarker of this disease [31–33]. However, COOH-terminal, mid-region, or whole structure of α-Enolase are all non-specific to Hashimoto encephalopathy because they can be found in many infectious and other autoimmune disease such as SLE, rheumatoid arthritis, or *Streptococcus pneumoniae* infection [34]. Unfortunately, in our country this test still not available so none of our patient had been check for this antibody.

Because of its many varieties of clinical presentation with non-specific findings of lab, MRI brain imaging, and CSF, diagnosis of Hashimoto encephalopathy is challenging. The key diagnostic clue can be depended on thyroid antibodies, but interpretation need to be cautious because their specificity to Hashimoto encephalopathy is not much that high and some literature also question about their pathogenic role [9]. Prevalence of these thyroid antibodies in general healthy population is 2 to 20% and as shown in Fig. 1, our patients who had positive thyroid antibodies were diagnosed with other conditions around 50–70%. So, there are many differential diagnoses to be ruled out first before suspicious of Hashimoto encephalopathy. Patients who presented with fever and alteration of consciousness are possibly due to central nervous system (CNS) infection which is more common than Hashimoto encephalopathy or if patients had pleocytosis in CSF, it can also from CNS infection or autoimmune limbic encephalitis [35]. Postictal phase of seizure or non-convulsive status epilepticus can be presented with encephalopathy [36] and Hashimoto encephalopathy also presented with seizure too, so complete evaluation by blood test, CSF study, brain imaging, and electroencephalography (EEG) is mandatory before diagnosed patient with Hashimoto encephalopathy. In our study, we do the grand rounds meeting by many subspecialty in neurology (e.g., epileptologist, behavioral neurologist, movement disorder specialist, stroke specialist, autoimmune neurology specialist) before getting the conclusive diagnosis of Hashimoto encephalopathy.

In clinical practice, blood test and EEG can be done easier than MRI and CSF examination so, most common cause of patient presented with encephalopathy will be ruled out first, such as delirium or seizure. MRI brain imaging may need time before doing due to its unavailability in some center but very important to do because it can show some hint of other disease that had worse prognosis than Hashimoto encephalopathy, for example, Creutzfeldt-Jakob Disease or metastasis brain tumor. If MRI brain imaging show enhancement in mesial temporal lobe, herpes encephalitis or autoimmune limbic encephalitis will be in differential diagnosis which prompt us to send virus polymerase chain reaction (PCR) or autoimmune encephalitis panel, both in blood and CSF, for evaluation. In our study, we sent both CSF and blood test for autoimmune and paraneoplastic encephalitis panel in all patients before diagnoses Hashimoto encephalopathy.

Even though we used so extensive evaluation and decision making by many subspecialties in neurology, there still can be error in diagnosis too as shown in recently publish article about misdiagnosis in autoimmune encephalitis in adults [37, 38]. Positive nonspecific serum antibody, such as antithyroid antibody, is one of red flag to consider about alternative diagnoses when meet patient presented with encephalopathy. So, diagnostic criteria of Hashimoto encephalopathy are mainly by exclusion of other possible causes (Table 3).

**Table 3:** Diagnostic criteria for Hashimoto encephalopathy^11^

1. Encephalopathy with seizures, myoclonus, hallucinations, or stroke-like episodes 2. Thyroid disease (subclinical or mild overt) 3. MRI scan of brain – normal or with nonspecific abnormalities 4. Serum thyroid antibodies present 5. Absence of other neuronal antibodies in serum or CSF 6. Exclusion of alternative causes of encephalopathy by differential diagnosis	

Regarding treatment outcomes in the present study, 1 patient who did not receive corticosteroid died from status epilepticus and septic shock. Of the remaining 12 patients who did receive corticosteroid treatment, 9 patients (75%) had marked improvement, 1 patient (8.3%) had slight improvement, and 2 patients (16.6%) had no clinical improvement. Those with clinical improvement had no difference in clinical features, laboratory findings, or neuroimaging findings compared to those without clinical improvement.

Previous studies [1, 9, 39] reported rates of favorable response to steroid treatment ranging from 36 to 93%, and our 75% rate of favorable response falls within that range. The wide range in the percentage of patient response to steroid therapy among studies may be due to differences in the duration of symptoms before the start of treatment, and differences in the study enrollment inclusion criteria. There are three articles that publish from our country about Hashimoto encephalopathy, two of them were case report [40, 41] and remaining one is prospective observation study [42]. In Charoensri A, et al.’s study [42] they had compared clinical between eleven false-positive antithyroid antibodies with six possible Hashimoto encephalopathy. Our study is different because we had more patients, both false-positive antibodies and Hashimoto encephalopathy, with more information about investigations results, and we also evaluate outcome of treatment. There is one study from Asian country, India, that study about clinical profile, radiological, and electrophysiological correlation to Hashimoto encephalopathy [43]. They show profiles and information like our study but they couldn’t do antibody testing of autoimmune encephalitis in every patient as ours, due to unavailability of test. They also didn’t explain about other differential diagnosis of 16 patients that they excluded.

### Limitations

This study has some mentionable limitations. First, our study’s retrospective design renders it vulnerable to missing or incomplete data and to certain biases. Second, due to the relative rarity of Hashimoto encephalopathy, our study sample size was small. Third, 82 (40%) patients who presented at our center with unexplained acute encephalopathy during the study period had no antibody testing data in their medical chart, so it is probable that we did not capture all cases of Hashimoto encephalopathy that occurred at our center during the study period. Fourth and last, we evaluated patients during only a five-year period (2012–2017), which may be not long enough for monitoring the emergence of clinical or laboratory manifestations of other autoimmune diseases. Another weakness of the short-term follow-up is the inability to evaluate the long-term prognosis and the relapse rate, which was previously reported to be 16% [9].

## Conclusion

The results of this study revealed cognitive impairment, neuropsychiatric symptoms, seizure, ataxia, and sleep disturbance to be common manifestations of Hashimoto encephalopathy. This condition should always be considered in individuals with subacute onset of unexplained cognitive impairment or cerebellar ataxia. Laboratory and neuroimaging investigations were all found to be nonspecific in Hashimoto encephalopathy. Most patients responded well to treatment, so clinical suspicion and early diagnosis and treatment will lead to improved patient outcomes.

### Electronic supplementary material

Below is the link to the electronic supplementary material.

Supplementary Material 1

## Data Availability

All data generated for this study are included in the article. There are no other datasets generated during the current study.

## List of Abbreviations

AMPAR: Alpha-amino-3-hydroxy-5-methyl-4-isoxazolepropionic acid receptor
CASPR2: Contactin-associated protein-like 2
CRMP5: Collapsing response mediator protein 5
GAD: Glutamic acid decarboxylase
Hu (ANNA-1): Antineuronal nuclear antibody 1
LGI1: Leucine-rich glioma inactivated 1
NAE: Amino (NH2)-Terminal of α-Enolase
NMDA: N-methyl-D-aspartate
Ri (ANNA-2): Antineuronal nuclear antibody 2
Yo (PCA1): Purkinje cell cytoplasmic antibody type 1

## References

[1] Castillo P, Woodruff B, Caselli R (2006). Steroid-responsive encephalopathy associated with autoimmune thyroiditis. Arch neurol.

[2] Kothbauer-Margreiter I, Sturzenegger M, Komor J (1996). Encephalopathy associated with Hashimoto thyroiditis: diagnosis and treatment. J Neurol.

[3] Afshari M, Afshari ZS, Schuele SU (2012). Pearls & oy-sters: Hashimoto encephalopathy. Neurology.

[4] Matsunaga A, Ikawa M, FujiiA (2013). Hashimoto’s encephalopathy as a treatable adult-onset cerebellar ataxia mimicking spinocerebellar degeneration. Eur Neurol.

[5] Schiess N, Pardo CA (2008). Hashimoto’s encephalopathy. Ann NY Acad Sci.

[6] Kirshner HS (2014). Hashimoto’s Encephalopathy: a brief review. Curr Neurol Neurosci Rep.

[7] Waliszewska-Prosół M, Ejma M. Hashimoto Encephalopathy-Still more questions than answers. Cells 2022 Sep 14;11(18):2873. 10.3390/cells11182873 PMC9496753 36139446

[8] Chaudhuri J, Mukherjee A, Chakravarty A (2023). Hashimoto’s Encephalopathy: Case Series and Literature Review. Curr Neurol Neurosci Rep.

[9] Laurent C, Capron J, Quillerou B (2016). Steroid-responsive encephalopathy associated with autoimmune thyroiditis (SREAT): characteristics, treatment and outcome in 251 cases from the literature. Autoimmun Rev.

[10] Marshall GA, Doyle JJ (2006). Long-term treatment of Hashimoto’s encephalopathy. J Neuropsychiatry Clin Neurosci.

[11] Chaudhuri A, Behan PO (2003). The clinical spectrum, diagnosis, pathogenesis and treatment of Hashimoto’s encephalopathy (recurrent acute disseminated encephalomyelitis). Curr Med Chem.

[12] Teasdale G, Jennett B (1974). Assessment of coma and impaired consciousness. A practical scale. Lancet.

[13] Wei LA, Fearing MA, Sternberg EJ (2008). The confusion Assessment Method: a systematic review of current usage. J Am Geriatr Soc.

[14] Graus F, Titulaer MJ, Balu R (2016). A clinical approach to diagnosis of autoimmune encephalitis. Lancet Neurol.

[15] Chong JY, Rowland LP, Utiger RD (2003). Hashimoto encephalopathy: syndrome or myth?. Arch Neurol.

[16] Mattozzi S, Sabater L, Escudero D (2020). Hashimoto encephalopathy in the 21st century. Neurology.

[17] Sliwinska A, Fumuso P, Stringer B (2020). Hashimoto Encephalopathy With Status Epilepticus Cureus.

[18] Guasp M, Giné-Servén E, Maudes E (2021). Clinical, neuroimmunologic, and CSF investigations in First Episode Psychosis. Neurology.

[19] Kayser MS, Titulaer MJ, Gresa-Arribas N (2013). Frequency and characteristics of isolated psychiatric episodes in anti-N-methyl-d-aspartate receptor encephalitis. JAMA Neurol.

[20] Höftberger R, van Sonderen A, Leypoldt F (2015). Encephalitis and AMPA receptor antibodies: novel findings in a case series of 22 patients. Neurology.

[21] Lopez-Chiriboga AS, Komorowski L, Kümpfel T (2016). Metabotropic glutamate receptor type 1 autoimmunity: clinical features and treatment outcomes. Neurology.

[22] Shaw PJ, Walls TJ, Newman PK (1991). Hashimoto’s encephalopathy: a steroid-responsive disorder associated with high anti-thyroid antibody titers—report of 5 cases. Neurology.

[23] Alazzeh A, Jaroudi S, Gooch M et al. Focal neurological presentation in Hashimoto’s encephalopathy mimicking a vascular occlusion of the middle cerebral artery.BMJ Case Rep 2017; 2017:bcr-2017-219933. 10.1136/bcr-2017-219933 PMC5534796 28710237

[24] Graham BR, Shiff N, Nour M (2016). Hashimoto Encephalopathy presenting with Stroke-Like episodes in an adolescent female: a case report and literature review. Pediatr Neurol.

[25] Valencia-Sanchez C, Pittock SJ, Mead-Harvey C (2021). Brain dysfunction and thyroid antibodies: autoimmune diagnosis and misdiagnosis. Brain Commun.

[26] Ferracci F, Carnevale A (2006). The neurological disorder associated with thyroid autoimmunity. J Neurol.

[27] Zhu Y, Yang H, Xiao F (2015). Hashimoto’s encephalopathy: a report of three cases and relevant literature reviews. Int J Clin Exp Med.

[28] Kelley BP, Patel SC, Marin HL (2017). Autoimmune encephalitis: pathophysiology and imaging review of an overlooked diagnosis. AJNR Am J Neuroradiol.

[29] Mocellin R, Walterfang M, Velakoulis D (2007). Hashimoto’s encephalopathy: epidemiology, pathogenesis and management. CNS Drugs.

[30] Jegatheeswaran V, Chan M, Chen YA (2021). MRI findings of two patients with Hashimoto Encephalopathy. Cureus.

[31] Ochi H, Horiuchi I, Araki N (2002). Proteomic analysis of human brain identifies alpha-enolase as a novel autoantigen in Hashimoto encephalopathy. FEBS Lett.

[32] Fujii A, Yoneda M, Ito T (2005). Autoantibodies against the amino terminal of α-enolase are a useful diagnostic marker of Hashimoto encephalopathy. J Neuroimmunol.

[33] Yoneda M, Fujii A, Ito A (2007). High prevalence of serum autoantibodies against the amino terminal of alpha-enolase in Hashimoto encephalopathy. J Neuroimmunol.

[34] Terrier B, Degand N, Guilpain P (2007). Alpha-enolase: a target of antibodies in infectious and autoimmune diseases. Autoimmun Rev.

[35] Sechi E, Flanagan EP (2021). Antibody-mediated Autoimmune Diseases of the CNS: Challenges and Approaches to diagnosis and management. Front Neurol.

[36] Dupont S (2019). Non convulsive status epilepticus in the elderly. Geriatr Psychol Neuropsychiatr Vieil.

[37] Flanagan EP, Geschwind MD, Lopez-Chiriboga AS (2023). Autoimmune encephalitis misdiagnosis in adults. JAMA Neurol.

[38] Valencia-Sanchez C, Pittock SJ, Mead-Harvey C (2021). Brain dysfunction and thyroid antibodies: autoimmune diagnosis and misdiagnosis. Brain Commun.

[39] Litmeier S, Pruss H, Witsch E (2016). Initial serum thyroid peroxidase antibodies and long-term outcomes in SREAT. Acta Neurol Scand.

[40] Riangwiwat T, Sangtian J, Sriphrapradang C. Steroid-responsive encephalopathy: an under recognized aspect of Hashimoto’s thyroiditis. Case Reports 2015; 2015: bcr2014208969. 10.1136/bcr-2014-208969 PMC4368923 25766444

[41] Termsarasab P, Pitakpatapee Y, Frucht SJ et al. Steroid-responsive Encephalopathy Associated with Autoimmune Thyroiditis (SREAT) presenting with pure cerebellar Ataxia. Tremor Other Hyperkinet Mov (N Y) 2018; 8: 585. 10.7916/D8CZ4QQQ PMC6125737 30191089

[42] Charoensri A, Tunlayadechanont S, Apiwattanakul M (2021). Autoimmune antibody in encephalopathic patients: a pilot study. J Med Assoc Thai.

[43] Sharma PM, Javali M, Mahale R (2015). Hashimoto encephalopathy: a study of the clinical profile, radiological and electrophysiological correlation in a Tertiary Care Center in South India. J Neurosci Rural Pract.

