# Supplementary material for: Clinical presentations and treatment outcomes of Hashimoto encephalopathy at Siriraj Hospital – Thailand’s largest national tertiary referral center
Source: BMC Neurol. 2023 Sep 22;23:334. doi: 10.1186/s12883-023-03305-4 (PMC10514970; doi:10.1186/s12883-023-03305-4)
Supplement: Supplementary file 1 — Supplementary Material 1 [file 12883_2023_3305_MOESM1_ESM.docx]

**Supplementary data**

Details of patients in our research.

| No. | Gender | Age (years) | Clinical features of encephalopathy | Neuropsychiatric symptoms | Thyroid status | Serum anti-TPO | Serum anti-Tg | Treatment outcome |
| --- | --- | --- | --- | --- | --- | --- | --- | --- |
| 1 | Male | 50 | Cognitive impairment | - | Euthyroid | + | + | Marked improve |
| 2 | Male | 42 | Clouding of consciousness, cognitive impairment | Delusion, mood disturbance | Euthyroid | + |  | No improvement |
| 3 | Male | 65 | Clouding of consciousness, cognitive impairment, behavioral change | - | Subclinical hyperthyroid |  | + | Slightly improve |
| 4 | Male | 58 | Behavioral change | Visual and auditory hallucination, mood disturbance | Subclinical hyperthyroid |  | + | Marked improve |
| 5 | Male | 61 | Clouding of consciousness, cognitive impairment | - | Euthyroid | + | + | Marked improve |
| 6 | Female | 74 | Clouding of consciousness | Mood disturbance | Euthyroid | + |  | Marked improve |
| 7 | Male | 75 | Cognitive impairment | Visual hallucination | Subclinical hypothyroid |  | + | Marked improve |
| 8 | Female | 58 | Cognitive impairment | - | Subclinical hypothyroid | + | + | Marked improve |
| 9 | Male | 64 | Clouding of consciousness | - | Subclinical hypothyroid | + | + | Marked improve |
| 10 | Female | 73 | Cognitive impairment | Visual and auditory hallucination | Subclinical hypothyroid | + | + | Marked improve |
| 11 | Male | 36 | Clouding of consciousness, Cognitive impairment | - | Euthyroid | + | + | Marked improve |
| 12 | Female | 73 | Cognitive impairment, behavioral change | Visual hallucination | Euthyroid | + |  | Death |
| 13 | Male | 24 | Cognitive impairment, behavioral change | - | Euthyroid |  | + | No improvement |

**Abbreviations:** TPO, thyroperoxidase; Tg, thyroglobulin
